# Attenuation of Redox Interference in RuO_2_ pH Sensor Using a Ta_2_O_5_/Nafion Multilayer Architecture

**DOI:** 10.3390/s26154960

**Published:** 2026-08-05

**Authors:** Wade Lonsdale, Magdalena Wajrak, Md Mahamudul Hassan, James Jin Kang

**Affiliations:** 1 School of Science, Edith Cowan University, Joondalup, Perth, WA 6027, Australia; w.lonsdale@ecu.edu.au (W.L.); mdmahamudul.hassan@ecu.edu.au (M.M.H.); 2 School of Science, Engineering and Technology, RMIT University, Ho Chi Minh City 700000, Vietnam; james.kang@rmit.edu.vn

**Keywords:** solid-state pH sensor, redox interference, RuO_2_ electrode, Ta_2_O_5_/Nafion composite layers, beverage samples

## Abstract

**Highlights:**

**What are the main findings?**

- Ta_2_O_5_ layers effectively suppress dissolved oxygen interference in RuO_2_ pH electrodes, while Nafion coatings significantly minimise interference from significant redox-active species such as ascorbic acid and permanganate.
- A combined Ta_2_O_5_/Nafion multilayer architecture delivers near-Nernstian sensitivity, low drift, and improved stability in RuO_2_-based pH sensors without severely compromising response performance.

**What are the implications of the main findings?**

- Multilayer barrier engineering provides a practical pathway to extend solid-state RuO_2_ pH sensors from controlled laboratory conditions into complex real-world matrices such as various beverages.
- The combined use of inorganic (Ta_2_O_5_) and polymeric (Nafion) coatings offers a design strategy for improving reliability and accuracy in electrochemical sensing platforms exposed to redox-active environments.

**Abstract:**

Despite the growing preference for solid-state pH sensors due to their advantages over glass pH electrodes, their practical application is still limited by challenges, particularly in metal-oxide based systems. The main issue is their susceptibility to redox-active species such as dissolved oxygen, ascorbic acid, sulfides, and transition-metal ions. In this work, the effects of representative redox interferents, with particular emphasis on ascorbic acid, together with dissolved oxygen and potassium permanganate, were investigated and mitigated through the application of Ta_2_O_5_/Nafion overlayers. The solid-state metal-oxide pH-sensitive electrodes were fabricated with laser micro-etching of radio-frequency (RF) sputtered RuO_2_ thin films deposited on ceramic Al_2_O_3_ substrates. The resulting RuO_2_ electrodes exhibited excellent potentiometric pH sensing characteristics, including near-Nernstian sensitivity (58.8 mV pH^−1^ at 25 °C), highly linear response over a wide pH range (pH 2–12, R^2^ > 0.9999), minimal hysteresis (1.3 mV), low potential drift (2.9 mV h^−1^), and fast response times (<30 s). Following on from our previous work, which demonstrated that the Ta_2_O_5_/Nafion solid-state sensor could measure beverage pH accurately, here we explain why that architecture works by systematically investigating the role of Ta_2_O_5_ and Nafion in suppressing redox interference and improving measurement stability in complex sample matrices. The results demonstrate that RuO_2_ electrodes modified with combined Ta_2_O_5_/Nafion thin films exhibit significantly enhanced measurement stability compared to unmodified RuO_2_ electrodes, owing to effective suppression of potential fluctuations induced by dissolved oxygen and representative redox couples, particularly those associated with ascorbic acid interference. These findings could give a promising strategy for improving the robustness and reliability of solid-state RuO_2_-based pH sensors in complex electrochemical environments.

## 1. Introduction: Challenges of Solid-State pH Sensors

Solid-state pH sensors were first introduced in 1970 [1]. There are many types of solid-state pH sensors, such as ion-sensitive field effect transistor sensors (ISFET) [2,3] and metal/metal-oxide electrodes like IrO_2_ or RuO_2_ [4,5,6,7,8]. However, despite the many advantages of the solid-state pH sensors, such as mechanical robustness, durability, miniaturisation, integration capabilities, faster response time, no requirement for an electrolyte, broader operating conditions, easier sterilisation, biocompatibility, smart options and ease of mass production, they still exhibit signal drift (especially ISFETs), require frequent calibration and can be sensitive to surface fouling or light (for semiconductor devices).

### 1.1. Redox Interference from Electroactive Species

Despite significant progress accomplished in solid-state pH sensors, their reliable deployment in complex chemical metrics such as biological fluids, beverages, environmental waters and industrial process streams remains challenging. One of the major challenges is to overcome the effect of non-pH-related shifts caused by the presence of redox-active species such as dissolved oxygen, ascorbic acid, sulfides, and transitional metal ions. The redox-active species participate in faradic reactions at the electrode surface and affect the accuracy and stability of the sensor [9,10,11,12].

Although metal-oxide-based potentiometric sensors, including RuO_2_, IrO_2_, and TiN, offer attractive advantages over conventional glass electrodes, as stated above, these sensors often exhibit performance degradation when exposed to real-world sample matrices containing multiple electroactive species, high ionic strength, organic compounds, and biological matter.

RuO_2_, while exhibiting near-Nernstian sensitivity and excellent stability in buffered laboratory solutions, is especially prone to such interference due to its high electrochemical activity. Similar challenges have been reported for IrO_2_ electrodes, which can exhibit large potential shifts in strongly oxidising or reducing environments. These limitations have motivated extensive research into material modification strategies that decouple proton sensitivity from parasitic redox processes [8,13]. However, while several studies have investigated interference from species such as dissolved oxygen and ferri/ferrocyanide, no study has systematically addressed ascorbic acid interference in metal-oxide pH sensors.

### 1.2. Drift, Hysteresis, and Long-Term Stability

Potential drift and hysteresis remain persistent challenges limiting the long-term reliability of solid-state pH sensors. Drift is commonly attributed to slow changes in surface hydration, rearrangement of oxygen vacancies, gradual surface reconstruction, and the irreversible adsorption of chemical species [14,15]. In complex environments, these effects are exacerbated by variations in ionic strength, temperature, and redox potential. Hysteresis, observed during cyclic pH measurements, arises from sluggish proton diffusion and incomplete equilibration of surface states, particularly in thick or porous oxide films [14,16]. In applications requiring continuous or repetitive measurements, such as process monitoring or wearable sensing, even small amounts of drift and hysteresis can lead to significant cumulative errors. Addressing these phenomena remains a key obstacle to replacing glass electrodes in real-world applications.

### 1.3. Environmental Monitoring

In environmental monitoring applications, redox-active species are frequently encountered and can compromise the accuracy of metal-oxide pH sensors. Dissolved oxygen concentrations vary naturally among well-aerated surface waters, groundwater, wastewater treatment systems, sediments, and biofilms, potentially introducing non-pH-related potential shifts. Natural organic matter and biologically derived reducing agents, including compounds analogous to ascorbic acid, are also common in environmental waters, while transition metal ions and oxidising species may be present in industrial effluents, mining-impacted waters, and contaminated sites. These constituents can alter the oxidation state of metal-oxide sensing surfaces and therefore interfere with potentiometric pH measurements. Consequently, the development of protective barrier layers capable of suppressing redox interference while maintaining proton transport is of significant importance for improving the reliability of solid-state pH sensors in long-term environmental monitoring applications, including surface-water quality assessment, wastewater treatment, aquatic ecosystem studies, and in situ monitoring networks.

### 1.4. Biofouling and Organic Contamination

In biological and environmental matrices, biofouling poses an additional, often underestimated, challenge. Proteins, polysaccharides, microorganisms, and other organic macromolecules can adsorb onto the sensor surface, altering interfacial charge transfer and proton exchange kinetics. Biofouling typically results in reduced sensitivity, increased response time, and poor calibration stability [17]. This issue is particularly critical for implantable sensors, wearable devices, and long-term environmental monitoring platforms, where frequent recalibration or sensor replacement is impractical.

### 1.5. Influence of Ionic Strength and Matrix Composition

While many solid-state pH sensors demonstrate excellent performance in simple buffer solutions, their responses can deviate significantly in samples with high ionic strength or complex electrolyte composition. Changes in ion activity coefficients, double-layer structure, and surface adsorption equilibria can disrupt the ideal Nernstian relationship between electrode potential and pH [18]. Additionally, matrix-dependent effects complicate calibration transferability between laboratory standards and real samples, further limiting practical applicability.

### 1.6. Protective Layers

To address redox interference and surface contamination, protective and selective membrane layers have been extensively investigated. Materials such as Ta_2_O_5_, Nafion, polymer electrolytes, and hydrogels have been employed to act as diffusion barriers that preferentially transport protons while suppressing electron transfer from interfering redox species [19]. Ta_2_O_5_ is particularly attractive due to its high dielectric constant, chemical stability, and pH-sensitive behaviour, making it effective as an inner barrier layer [20]. Nafion, a perfluorosulfonic acid polymer, provides high proton conductivity and electrostatic exclusion of negatively charged redox species [19].

However, the introduction of protective layers inevitably introduces trade-offs. Increased membrane thickness and tortuosity can slow response times and attenuate sensitivity. Achieving an optimal balance between selectivity, stability, and dynamic response remains a key design challenge. Multilayer architectures combining inorganic oxides (e.g., Ta_2_O_5_) with polymeric membranes (e.g., Nafion) have emerged as a promising strategy to mitigate these competing effects [21].

## 2. Solid Metal-Oxides Electrodes Theory

The use of metal-oxides for the development of solid-state potentiometric pH sensors has been reported in the literature for several decades [22,23,24]. Of the various metal-oxide pH sensors reported, IrO_2_ is one of the most commonly studied materials due to its chemical stability and excellent performance [4,8,25]. RuO_2_ has emerged as an attractive alternative to IrO_2_ due to its lower cost and comparable performance characteristics [26,27]. In solution, RuO_2_ hydrates, resulting in the following reversible redox couple [28,29].

(1) $${RuO}_{x}{\left(OH\right)}_{y}+ze^{-}+{zH}^{+}↔{RuO}_{x-y}{\left(OH\right)}_{y+z}$$

which simplifies to

(2) $${Ru}^{\left(IV\right)}O_{2}+e^{-}+H^{+}↔{Ru}^{\left(III\right)}O(OH)$$

where the potential of an electrode is given by the Nernst equation:

(3) $$E=E^{0}-\frac{RT}{F}ln\frac{a\left[{Ru}^{III}\right]}{a\left[{Ru}^{IV}\right]\left[H^{+}\right]}=\left(E^{0}-\frac{RT}{F}ln\frac{a[{Ru}^{III}]}{a[{Ru}^{IV}]}\right)-\frac{RT}{F}ln\left(a[H^{+}]\right)$$

where E^0^—standard potential, R—universal gas constant, T—absolute temperature, F—Faraday constant and *a*[Ru^III^], *a*[Ru^IV^], and *a*[H^+^]—activity of Ru^III^, Ru^IV^, and H^+^, respectively. At equilibrium, the electrode establishes a stable Ru^3+^/Ru^4+^ ratio, and Equation (3) can therefore be simplified to

(4) $$E=E^{*}-58.6pH$$

where E is the measured potential in mV at 22 °C and E* represents the electrode’s potential at a pH of 0.

In order to make accurate measurements, it is crucial that the Ru^III^/Ru^IV^ ratio (and therefore the E* value) remains constant [26]. As a result of this, metal-oxide pH sensors only operate accurately in sample matrices free from oxidising or reducing agents, such as ferri/ferro-cyanide, iodide, permanganate, dissolved oxygen and ascorbic acid [11,26]. Such “redox-active” species donate or accept electrons with RuO_2_, resulting in the oxidisation or reduction in the electrode and therefore a shift in the E* value [26]. As a result, the application of metal-oxide pH sensors is largely limited to relatively simple sample matrices, including fresh surface waters [30], and to applications where stringent accuracy requirements are not necessary, such as acid reflux detection [25]. The successful deployment of these sensors in more complex matrices requires the prevention of electron transfer between the metal-oxide surface and the sample solution, while preserving proton transport to the electrode surface [11,31]. Kinlen et al. [11] demonstrated that redox interference in metal-oxide pH sensors can be partially suppressed through the application of a thermally cured Nafion coating. The cured Nafion layer was found to prevent interference from ferri/ferrocyanide and to reduce interference arising from I^−^ and MnO_4_^−^ ions. Although Nafion has also been utilised in other studies [32,33], it was applied without thermal curing and served primarily as a protective coating to improve the durability of the metal oxide by minimising delamination and dissolution [34,35]. Although Nafion has exhibited promising qualities in terms of reducing or possibly eliminating redox interference, it is known to swell due to the uptake of water. The absorbance of water is essential for the conduction of protons; however, swelling can result in the degradation and eventual delamination of the Nafion membrane [36].

An alternative approach to reducing redox interference in metal-oxide pH sensors was proposed by Kuo et al. [37]. The authors coated a pH-sensitive IrO_2_ electrode with a 76 nm layer of Ta_2_O_5_, a proton-conducting and electrically insulating material. This coating was shown to suppress electron transfer arising from dissolved oxygen while maintaining pH sensitivity. Although the authors concluded that the Ta_2_O_5_ layer eliminated interference from redox-active species, this effect was demonstrated only for dissolved oxygen. Protection from dissolved oxygen is advantageous for a pH sensor, as it allows for measurements between aerobic/anaerobic conditions, without the need for recalibration, and helps improve the stability due to the elimination of potential fluctuations caused by dissolved O_2_ [37]. However, protection from other redox species is more important, if one aims to utilise metal-oxide pH sensors in applications with more complex sample matrices. Ascorbic acid is a common agent in many samples, such as beverages (including white wine, orange and other citrus juices) and biological samples such as blood and gastric fluid. Therefore, the aim of this study was to investigate Nafion and Ta_2_O_5_ protective layers over a RuO_2_ electrode in order to mitigate redox interference from ascorbic acid and other species such as potassium permanganate and dissolved oxygen.

## 3. Methodology

### 3.1. RuO_2_ Electrode Fabrication

**Figure 1:**
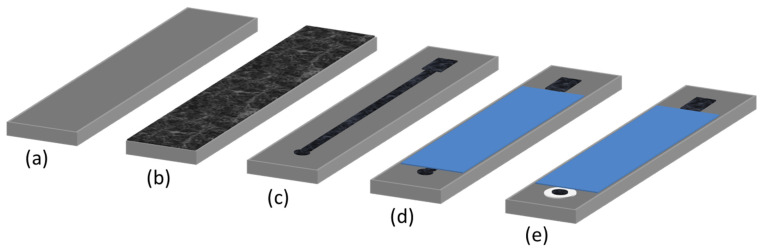
Manufacture of working electrodes: (**a**) Al_2_O_3_ substrate, (**b**) 500 nm RuO_2_, (**c**) laser etch pattern, (**d**) electrical isolation with resin, and (**e**) working area modification.

A 500 nm thick RuO_2_ film was deposited onto a 1 mm-thick Al_2_O_3_ substrate to fabricate the pH-sensitive working electrodes. The film was deposited by radio-frequency magnetron sputtering (RFMS) from a 99.95% purity RuO_2_ target at room temperature, using a sputtering power of 100 W, an Ar:O_2_ process gas ratio of 1:9, and a chamber pressure of 4 mTorr. These deposition parameters have previously been shown to produce a RuO_2_ film thickness of approximately 500 nm [26]. The sensing area, conductive track, and electrical contact pad were then defined by laser patterning of the RuO_2_ layer using a Speedy 360 Flex Trotec laser cutter/engraver (Trotec, Heinsberg, Germany, 75% power, 8% speed, four passes). Finally, Gwent dielectric paste was applied and cured at 120 °C for 20 min to electrically isolate the sensing area, as shown in Figure 1.

### 3.2. Electrode Modification

To evaluate the effects of insulating surface layers, the RuO_2_ electrodes were modified with either Ta_2_O_5_ or Nafion coatings. Ta_2_O_5_ films with thicknesses of 150 and 500 nm were deposited by radio-frequency magnetron sputtering (RFMS) from a 99.95% purity Ta_2_O_5_ target at room temperature using a sputtering power of 200 W, an Ar:O_2_ process gas ratio of 1:9, and a chamber pressure of 4 mTorr.

Nafion coatings of two nominal thicknesses were also investigated. Thin coatings were prepared by manually dip-coating the electrodes three times in a 5% Nafion solution (Sigma, St. Louis, MO, USA), while thick coatings were produced by drop-casting 50 μL of the solution onto the sensing area. The exact film thicknesses were not measured, as the objective was to compare relatively thin and thick Nafion layers. Nevertheless, dip-coating is expected to yield films ranging from approximately 100 nm to several micrometres in thickness, whereas drop-casting 50 μL of Nafion typically produces coatings on the order of 1–20 μm.

After deposition, the Nafion-coated electrodes were thermally cured by rapid thermal annealing (RTA) at 230 °C for 15 min under vacuum (<10 mTorr). These modifications, together with the uncoated controls, yielded a total of eight electrode configurations, as shown in Table 1.

### 3.3. Potentiometric Measurements

The electrochemical potential of each fabricated working electrode was measured against a double-junction Ag/AgCl|KCl reference electrode (Sigma) using an Agilent 34410A high-performance digital multimeter (Santa Clara, CA, USA). Potentials were recorded for 300 s at 1 s intervals with the instrument operating in high-impedance mode and an NPLC setting of one. To minimise the influence of transient effects, the electrode potential for each measurement was taken as the average of the final 30 recorded data points. These values were used to determine sensor sensitivity, E^0^, hysteresis, and drift. Hysteresis was calculated from the difference between consecutive measurements at pH 7, while drift was determined from the slope of the linear best-fit of the pH 7 potential-time data. Sensor response time was defined as the time required for the potential to reach within 3 mV (corresponding to 0.05 pH units) of its steady-state value. Measurements were conducted at 22 °C in commercial pH buffer solutions (Rowe Scientific, Wangara WA, Australia), and all error bars represent 95% confidence intervals.

### 3.4. pH Sensing Measurements

Interference studies were performed using representative reducing and oxidising agents, namely ascorbic acid and potassium permanganate, respectively. Fresh 1 mM stock solutions were prepared daily by dissolving analytical-grade L-ascorbic acid (Merck, Rahway, NJ, USA) and potassium permanganate (Merck) in deionised water. The solutions were protected from light and used within the day of preparation to minimise decomposition, particularly of ascorbic acid. Ascorbic acid and potassium permanganate are commonly used in electrochemical sensor studies as model reducing and oxidising species for evaluating redox susceptibility of potentiometric electrodes. For interference testing, electrodes were initially equilibrated in commercial pH 4 buffer solution. Following stabilisation of the electrode potential, an aliquot of the appropriate stock solution was added to the buffer under gentle stirring to obtain a final interferent concentration of 1 mM. The resulting potential was recorded continuously for 5 min using the procedure described in Section 3.3. The magnitude of redox interference was quantified as the difference between the stable potential before addition of the interferent and the potential measured after 5 min exposure. Similar approaches have been reported previously for evaluating the influence of oxidising and reducing species on metal-oxide pH sensors. Commercial beverage samples, including cola, beer, iced tea, sports drink, milk, whiskey, vinegar, orange fruit drink, fresh orange juice and white wine, were purchased from local retailers and analysed as received. No dilution, pH adjustment or addition of redox-active reagents was performed. Electrodes were first equilibrated in pH 4 buffer, after which they were immersed directly into the beverage sample. The change in potential relative to the initial buffer potential was monitored for 5 min and reported as the beverage-induced potential shift. This methodology was selected to assess the effect of naturally occurring redox-active constituents present in real sample matrices on sensor performance.

Ascorbic acid and potassium permanganate were selected as representative reducing and oxidising redox-active species, respectively. A concentration of 1 mM was chosen as it provided measurable and reproducible potential perturbations suitable for comparing the effectiveness of the various protective-layer architectures. The purpose of these experiments was to evaluate electrode susceptibility to representative electroactive interferents rather than to replicate the exact composition of any specific sample matrix. Although ascorbic acid may also influence the solution pH owing to its weakly acidic nature, the observed response is expected to arise predominantly from its interaction with the RuO_2_ redox surface, which is known to be sensitive to oxidising and reducing agents.

**Figure 2:**
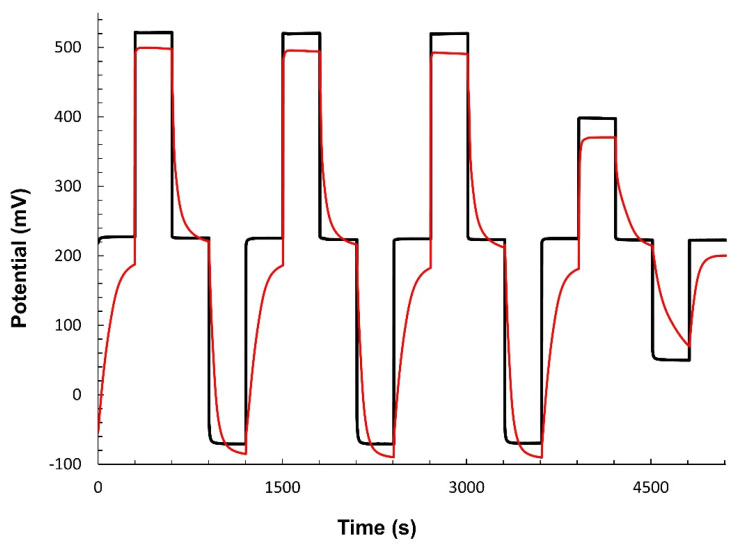
The 500 nm RuO_2_ (Black) and 500 nm RuO_2_ + 50 µL Nafion (Red) electrodes from pH 7-2-7-12 three times, followed by pH 7-4-7-10-7.

**Figure 3:**
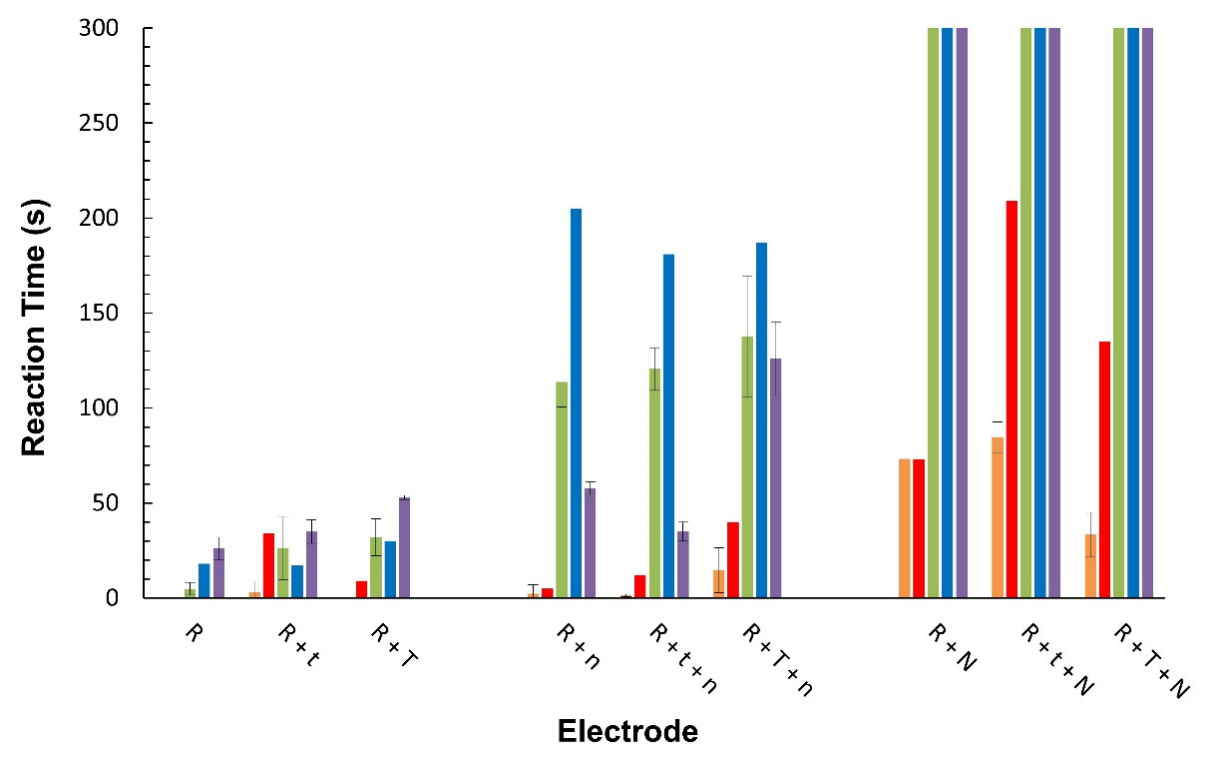
Reaction times for developed electrodes at pH 2 (orange), 4 (red), 7 (green), 10 (blue) and 12 (purple). Note: Bars that reach 300 s are much greater than this, in the order of several hours.

Prior to use, electrodes were conditioned by boiling in deionised water for 30 min, then equilibrated overnight in pH 7 buffer. The pH sensing performance for each electrode was evaluated by “looping” pH from 7-2-7-12 three times followed by 7-4-7-10-7 once, as shown in Figure 2 for the R and R + N electrodes. Table 2 summarises the pH sensing performance results for the different electrode modifications, whilst Figure 3 shows the electrode reaction times at the pH values tested.

## 4. Results and Discussion

### 4.1. pH Sensing Performance

The unmodified RuO_2_ electrode (R) exhibited excellent pH sensing performance, demonstrating near-Nernstian sensitivity (58.8 mV pH^−1^), a linear response over the pH range 2–12 (R^2^ > 0.9999), low hysteresis (1.3 mV), low drift (2.9 mV h^−1^), and acceptable response times (<30 s), as summarised in Table 2. These results are comparable to those previously reported for RuO_2_ electrodes fabricated on screen-printed carbon substrates [26].

The incorporation of 150 nm or 500 nm Ta_2_O_5_ coatings (R + t and R + T, respectively) did not adversely affect sensor performance. Sensitivity, linearity, hysteresis, drift, and response times remained comparable to those of the unmodified electrode, consistent with the observations of Kuo et al. [37].

In contrast, the addition of Nafion altered the pH sensing characteristics of the RuO_2_ electrodes. Thin Nafion coatings (electrodes denoted by ‘n’ in Table 1) improved both drift and hysteresis (Table 2); however, they increased the response time at neutral and alkaline pH values from approximately 30–60 s to 150–200 s (Figure 3). This behaviour is consistent with that reported by Kinlen et al. [11].

Thick Nafion coatings (electrodes denoted by ‘N’ in Table 1) produced a substantially greater increase in response time at pH 7, 10, and 12 (Figure 3). The actual response times for these electrodes were approximately 2–3 h. This behaviour is also evident in Figure 2, where the R + N electrode fails to reach equilibrium within the 5 min measurement period. Consequently, the linearity and E^0^ values reported in Table 2 for the thick Nafion-coated electrodes were calculated excluding the pH 10 and pH 12 measurements, although the response at pH 7 remained acceptable. Similarly, the large hysteresis values obtained for these electrodes are primarily attributable to incomplete equilibration during the measurement period rather than true hysteretic behaviour.

### 4.2. Redox Interference

**Figure 4:**
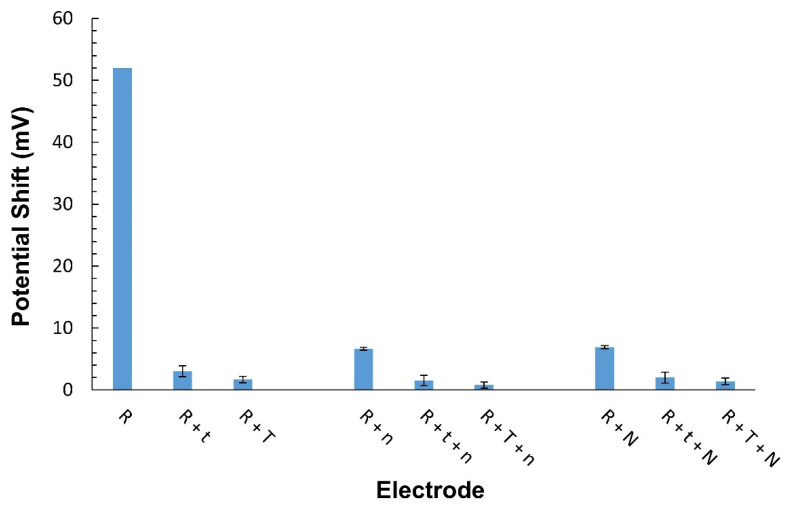
Potential shift after 2 h in oxygen saturated pH 4 buffer.

**Figure 5:**
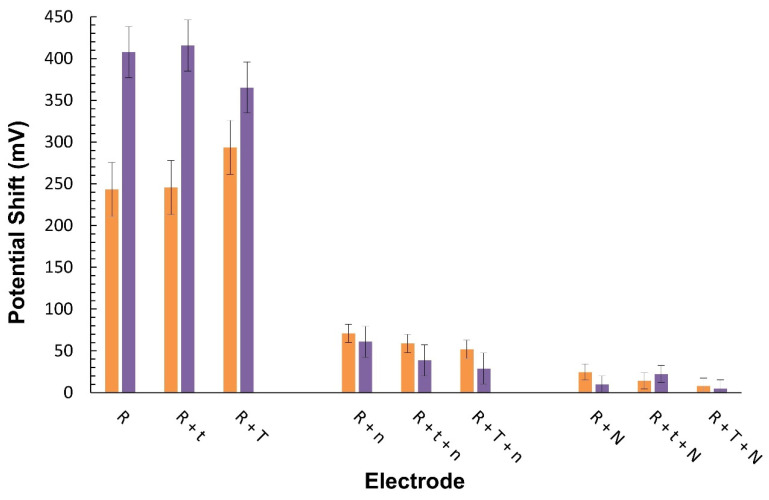
Potential shift after 5 min at pH 4 caused by the redox interference from 1 mM ascorbic acid (orange) and 1 mM KMnO_4_ (purple) for each of the electrodes.

Figure 4 illustrates the potential shifts induced by changing the dissolved gas environment from N_2_-saturated to O_2_-saturated pH 4 buffer, while Figure 5 shows the potential changes observed following the addition of 1 mM ascorbic acid or 1 mM KMnO_4_ to a pH 4 buffer.

As expected, the unmodified RuO_2_ electrode (R) exhibited substantial potential shifts in the presence of all three redox-active species (Figure 4 and Figure 5), confirming its susceptibility to redox interference. In agreement with the findings of Kuo et al. [37], the Ta_2_O_5_-modified electrodes (R + t and R + T) effectively eliminated interference arising from dissolved oxygen. As shown in Figure 4, the potential shifts observed for these electrodes were smaller than the drift values reported in Section 3.1, indicating that the influence of dissolved oxygen was negligible.

However, the Ta_2_O_5_ coatings were ineffective against stronger redox-active species. Significant potential shifts were still observed following the addition of ascorbic acid and KMnO_4_ (Figure 5), demonstrating that Ta_2_O_5_ alone does not provide sufficient protection against all forms of redox interference.

Nafion coatings also reduced the interference caused by dissolved oxygen, although complete suppression was not achieved (Figure 4, R + n and R + N). In addition, Nafion decreased the potential shifts induced by both ascorbic acid and KMnO_4_, with thicker Nafion coatings providing greater protection than thinner coatings. This trend is consistent with the observations of Kinlen et al. [11].

The combined results suggest that Ta_2_O_5_ and Nafion mitigate redox interference through complementary mechanisms. The Ta_2_O_5_ layer acts primarily as an electron-transfer barrier, effectively suppressing interference from dissolved oxygen while maintaining proton transport. In contrast, the Nafion overlayer enhances selectivity by facilitating proton conduction and electrostatically excluding negatively charged interferents. Together, these mechanisms contribute to improved resistance to redox interference and enhanced overall sensor selectivity.

### 4.3. Application to Beverage Samples

**Figure 6:**
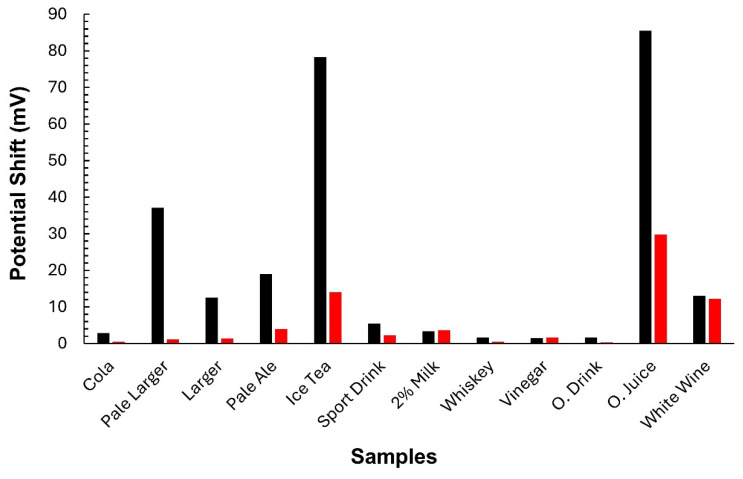
Potential shifts for R (black) and R + t + n (red) electrodes after 5 min of exposure to various sample matrices.

Although thicker Nafion coatings further reduced redox interference, they were not used for beverage measurements because the resulting increase in proton diffusion path length produced excessively slow equilibration times (typically several hours). Consequently, due to its excellent pH sensing performance, acceptable reaction time, and improvement in redox interference, the R + t + n electrode was selected for application to some sample matrices. Figure 6 shows the redox shift that occurred when an unprotected R electrode and an R + t + n electrode were exposed to some common beverages: cola, beer (pale lager, lager, pale ale), ice tea, sports drink, 2% fat milk, whiskey, vinegar, orange fruit drink (O. drink), fresh orange juice (O. juice) and white wine. Figure 6 shows that for the cola, sports drink, milk, vinegar and orange fruit drink samples, an unprotected R could be suitable; however, a modified R + t + n electrode would be more suitable, due to the lower potential shifts observed. The advantage of the R + t + n electrode is clearly visible for the beer samples, where a large shift was observed for the unprotected R electrode, but a minimal shift was observed for the R + t + n. It is also shown in Figure 6 that some matrices are still unsuitable for the R + t + n electrode (e.g., iced tea, fresh orange juice and wine). It is interesting to note the large difference in potential shift between the orange fruit drink and fresh orange juice samples, which would be due to ascorbic acid present in the fresh juice but not in the processed drink.

## 5. Conclusions

In this study, a series of modified solid-state RuO_2_ pH-sensitive electrodes were systematically developed and evaluated using Ta_2_O_5_ and dip-coated Nafion surface barrier layers to mitigate redox interference. The modified RuO_2_ electrodes demonstrated excellent intrinsic pH sensing performance, including near-Nernstian sensitivity (58.8 mV pH^−1^), highly linear response over a wide pH range (pH 2–12, R^2^ > 0.9999), low hysteresis (1.3 mV), low potential drift (2.9 mV h^−1^), and acceptable dynamic response times (<30 s).

Experimental investigations revealed that sputter-deposited Ta_2_O_5_ acts as an effective inorganic barrier against interference from dissolved oxygen, which is attributable to its chemical stability and limited electronic conductivity. However, Ta_2_O_5_ alone was insufficient to fully suppress potential perturbations induced by stronger redox-active species, such as ascorbic acid and permanganate ions. In contrast, Nafion coatings significantly attenuated interference arising from both oxidising and reducing agents, including dissolved oxygen, ascorbic acid, and permanganate, owing to its selective proton conductivity and electrostatic exclusion of anionic redox species. This improved selectivity was achieved at the expense of moderately increased response times, particularly in neutral to alkaline pH regimes, due to additional mass-transport limitations imposed by the polymeric membrane.

Importantly, the combined Ta_2_O_5_/Nafion multilayer architecture yielded a synergistic improvement in sensor performance. RuO_2_ electrodes modified with an inner Ta_2_O_5_ layer and an outer Nafion barrier exhibited substantially enhanced measurement stability and reduced potential fluctuations compared with unmodified RuO_2_ electrodes, while largely preserving favourable sensitivity and response characteristics.

Overall, the demonstrated Ta_2_O_5_/Nafion-modified RuO_2_ electrodes constitute a promising platform for accurate and robust pH monitoring in real-world applications, particularly in beverage analysis where redox-active constituents are commonly present, such as soft drinks, fruit drinks, beer, whiskey and milk, thus presenting a significant advancement toward improving the reliability of solid-state RuO_2_ pH sensors in chemically complex matrices.

The present study specifically focuses on mitigating redox interference in RuO_2_-based pH sensors using Ta_2_O_5_/Nafion barrier layers. While other factors affecting long-term sensor performance, including biofouling, organic contamination, ionic strength effects, and storage stability, are recognised as important challenges, comprehensive investigation of these phenomena is beyond the scope of the current work. However, this approach does offer a practical compromise between selectivity, stability, and response time, and provides a foundation for further optimisation of multilayer barrier designs for solid-state pH sensing in challenging environments.

## Figures and Tables

**Table 1:** Codes and structures of the different manufactured RuO_2_ electrodes.

Code	Electrode
R	500 nm RuO_2_
R + t	500 nm RuO_2_ + 150 nm Ta_2_O_5_
R + T	500 nm RuO_2_ + 500 nm Ta_2_O_5_
R + n	500 nm RuO_2_ + Dip Coated Nafion
R + t + n	500 nm RuO_2_ + 150 nm Ta_2_O_5_ + Dip Coated Nafion
R + T + n	500 nm RuO_2_ + 500 nm Ta_2_O_5_ + Dip Coated Nafion
R + N	500 nm RuO_2_ + 50 µL Nafion
R + t + N	500 nm RuO_2_ + 150 nm Ta_2_O_5_ + 50 µL Nafion
R + T + N	500 nm RuO_2_ + 500 nm Ta_2_O_5_ + 50 µL Nafion

**Table 2:** Summary of electrode performance. Note that results marked “*” were calculated without pH 10 or 12 values.

Code	Sensitivity (mV/pH)	E*	R^2^	Hysteresis (mV)	Drift (mV/h)
R	−58.8 ± 0.47	636 ± 4.0	0.9999	1.3 ± 0.5	2.9
R + t	−58.9 ± 0.86	670 ± 3.3	0.9995	1.8 ± 1.2	2.8
R + T	−58.3 ± 0.91	669 ± 11	0.9997	1.8 ± 1.1	8.2
R + n	−57.9 ± 0.49	606 ± 4.1	0.9998	0.54 ± 0.47	0.48
R + t + n	−58.5 ± 0.54	658 ± 1.1	0.9998	0.57 ± 0.29	0.92
R + T + n	−58.6 ± 1.1	744 ± 7	0.9994	3 ± 1.5	0.35
R + N	−58.7 ± 8.5 *	609 ± 10.6 *	0.9995	31.6 ± 4.8	2.4
R + t + N	−56.6 ± 0.89 *	661 ± 5.1 *	0.9971	56.1 ± 9.4	1.5
R + T + N	−59.5 ± 2.8 *	677 ± 1.4 *	0.9998	46.2 ± 8.7	5.2

## Data Availability

The original contributions presented in this study are included in the article. Further inquiries can be directed to the corresponding author(s).
